# FALL PREVENTION IN RURAL COMMUNITIES OF NORTH DAKOTA

**DOI:** 10.1093/geroni/igz038.3550

**Published:** 2019-11-08

**Authors:** Melissa L OConnor, Jane Strommen, Philip Estepp, Megan Hay, Heather Fuller, Rachel Grace, Sean Brotherson

**Affiliations:** North Dakota State University, Fargo, North Dakota, United States

## Abstract

Fall-related injuries are common sources of morbidity and mortality for adults aged 65 and older. Thus, interventions for preventing falls can have substantial public health benefits. One promising fall prevention program is the Stepping On program, which involves seven community-based workshops. In these workshops, older adults learn about risk factors for falls, as well as safety-conscious behaviors. Stepping On has been offered in several states, and outcomes have been positive. However, research in rural areas has been lacking. To address this issue, the current study examined 508 older adults who participated in the Stepping On program across 53 rural communities in North Dakota through May of 2019. Most participants were female (82%), with an average age of 79 years (range 65-98). Participants completed baseline and post-test assessments of their knowledge regarding health, mobility, and safety issues. At baseline, 46% of participants reported falling at least once during the previous year. Repeated-measures ANOVAs showed that participants had improved significantly in the following areas at post-test: understanding how vision influences safety; knowledge of balance and strength exercises; recognizing hazards in the home; choosing safe footwear; confidence in mobility; understanding how medications affect fall risk; and the importance of bone health (p<0.001 for all). Just 14% of participants reported falling during the year following the workshops, and 62% felt that the program reduced their risk of falls “to a big extent.” These findings suggest that the Stepping On program is feasible to administer in rural areas and benefits older adults in such communities.

